# A Robust Vehicle Localization Approach Based on GNSS/IMU/DMI/LiDAR Sensor Fusion for Autonomous Vehicles

**DOI:** 10.3390/s17092140

**Published:** 2017-09-18

**Authors:** Xiaoli Meng, Heng Wang, Bingbing Liu

**Affiliations:** 1 Institute for Infocomm Research, Agency for Science, Technology and Research (A*STAR), Singapore; xiaoli.meng09@gmail.com (X.M.); bliu@i2r.a-star.edu.sg (B.L.); 2 College of Information Science and Engineering, Northeastern University, Shenyang 110819, China

**Keywords:** sensor fusion, Unscented Kalman Filter (UKF), vehicle localization

## Abstract

Precise and robust localization in a large-scale outdoor environment is essential for an autonomous vehicle. In order to improve the performance of the fusion of GNSS (Global Navigation Satellite System)/IMU (Inertial Measurement Unit)/DMI (Distance-Measuring Instruments), a multi-constraint fault detection approach is proposed to smooth the vehicle locations in spite of GNSS jumps. Furthermore, the lateral localization error is compensated by the point cloud-based lateral localization method proposed in this paper. Experiment results have verified the algorithms proposed in this paper, which shows that the algorithms proposed in this paper are capable of providing precise and robust vehicle localization.

## 1. Introduction

Automated driving techniques are widely admitted as a promising and challenging way to avoid road crashes and improve traffic conditions [1]. To make an autonomous vehicle drive safely in urban environments, the vehicle needs to know its exact position and orientation. Thus, localization plays a key role in autonomous vehicle applications. Using the popular GNSS (Global Navigation Satellite System) for localization requires a GNSS receiver with an unobstructed line of sight to four or more GNSS satellites. However, even with a high-end GNSS-based system, a vehicle’s location may jump up to a few meters as different satellites go in and out of view or obstructions in the environments create multi-path interferences. INS (Inertial Navigation System) is complementary to GNSS as it does not rely on external information sources, which can be blocked or disturbed. INS can provide complete navigation information such as position, velocity and attitude by integrating the accelerometer and gyroscope readings over time. However, as the inertial sensors of an INS are subject to drifts, navigation systems based on stand-alone INS suffer a rapid degradation of position over time. This is particularly true when a low-cost IMU (Inertial Measurement Unit) is employed. In the dead-reckoning integration scheme, wheel encoders are always introduced to slow the rate of growth of IMU integration errors, but are subject to errors due to wheel slip. However, a robust localization solution can be achieved by blending GNSS, INS and DMI (Distance Measuring Instruments) techniques in a way that utilizes the strengths of each individual system and mitigates their weaknesses.

To fuse GNSS, INS and DMI, Extended Kalman Filtering (EKF) is a popular sensor fusion method [2,3], where nonlinear systems are linearized and approximated around current state estimates. However, in the EKF, high-order terms are neglected, which are necessary for some situations. The Particle Filter (PF) [4,5] is another useful method, but the main drawback of this filter is its computational requirement, which makes it not very suitable for real-time applications. In [6], an INS/GPS sensor fusion scheme based on the State-Dependent Riccati Equation (SDRE) nonlinear filtering method is proposed for Unmanned Aerial Vehicles (UAV), which is widely used in the optimal nonlinear control and filtering literature. Although this method provides an alternative INS/GPS filtering scheme, the real-time performance and application to autonomous vehicles in urban environments are still uncertain. In [7,8], low cost sensors such as cameras are fused with GNSS/INS to improve the localization accuracy of GNSS in dense urban areas where obstacles block satellite signals; however, the improvement of accuracy is limited due to the difficulties in some feature recognition tasks and the calculating of depth information using cameras. Recently, the Unscented Kalman Filter (UKF) has been used for localization based on GPS/INS sensor fusion [9,10,11] due to the ability to remove the messy Jacobian matrix computation and keep at least a second-order nonlinear function approximation. Although UKF has been proven to be a promising method for GPS/INS fusion, the accuracy and reliability performance still need to be improved for autonomous vehicles under urban environments.

On the other hand, the localization approach based solely on GNSS/IMU/DMI cannot always guarantee a precise location solution due to the existence of the blocking of satellite signals by obstacles (buildings and trees, etc.) and the cumulative errors of IMU and DMI sensors. In order to provide precise localization for positioning an autonomous vehicle reliably, we need to explore other useful information to position an autonomous vehicle. In the urban environment, curbs and lane markings comprise two kinds of useful information for improving the results of GPS/INS/DMI fusion. For example, in [12,13,14,15], cameras are fused with other sensors such as GPS and IMU to improve the lateral accuracy by detecting lane markings. However, the detection of lane marking needs to face the challenges of different lighting, poor lane markings, etc. On the other hand, 3D point clouds generated by a 3D LiDAR scanner provide more reliable performance for curb detection, which can also be used to improve the lateral localization accuracy [16,17,18,19,20]. Furthermore, in [21], an eigenvector technique was used to find a line segment corresponding to edges of roads. In [22], a Hough transform was used to find the best fit line to the surface on the road, and points corresponding to the best fit line were used as curb points of the road. In [23], curbs are extracted by using a 1D laser scanner.

**Figure 1:**
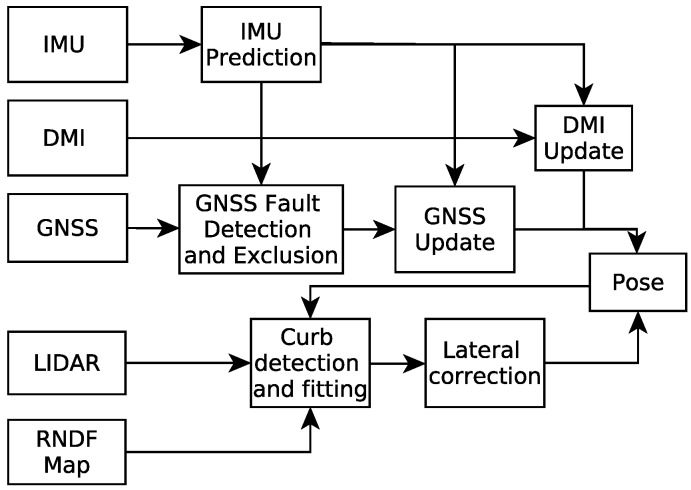
The flowchart of the proposed method. DMI, Distance-Measuring Instrument; RNDF, Route Network Definition File.

In this paper, to further improve the accuracy and reliability of localization for autonomous vehicles in urban environments, we firstly propose a fault-detection-based loosely-coupled GNSS/IMU/DMI localization solution, which can improve the performance of the traditional UKF-based method; then, we correct the lateral localization errors based on curb detection results using a multi-layer LiDAR. The flowchart of overall localization method is summarized in Figure 1.

The main contribution of this paper is summarized as follows. Firstly, through combining the fault-detection method with the UKF-based GNSS/IMU/DMI fusion algorithm, the localization accuracy of autonomous vehicles is improved greatly; Secondly, a point cloud-based curb detection and fitting method is proposed to improve the lateral accuracy of the autonomous vehicle further, where the RANSAC algorithm is utilized. The rest of the paper is organized as follows: Section 2 presents the proposed UKF-based localization approach: firstly, the modeling including the process model and the measurement model is introduced, followed by the implementation of the UKF. Details about curb-based lateral localization are provided in Section 3. Section 4 presents the experimental results. Finally, we conclude the paper in Section 5.

## 2. UKF-Based Localization Approach

For a vehicle localization system, four coordinate systems are defined:

- Earth-Centered-Earth-Fixed (ECEF) coordinate system (E): It has an origin at the center of the Earth. The positive Z-axis goes out the Earth’s north pole; the X-axis is along the prime meridian; and the Y-axis completes the right-handed system;
- Global coordinate system (G): The North-East-Down (NED) coordinate system is defined as G with the X-axis pointing north, the Y-axis pointing east and the Z-axis pointing down to construct a right-handed coordinate system;
- Body coordinate system (B): The coordinate system of the vehicle with the X-axis pointing forwards, the Y-axis pointing left and the Z-axis pointing up;
- Sensor coordinate system (S): the three orthogonal axes of the mounted sensors. We assume that S coincides with B after sensor to body alignment calibration [24].

One should note that each sensor defines its own coordinate system. We need to note the difference between the origins for accurate localizations.

We describe the states of the filtering system with the following vector:

(1) $$x={\left[p,v,q,b,d\right]}_{16\times 1}^{T}$$

where p and v are the position and velocity of the vehicle within the global frame G, respectively. q is a unit quaternion that represents the rotation from the body frame B to the global frame G. A unit quaternion consists of a vector part $e={\left(q_{1},q_{2},q_{3}\right)}^{T}\in R^{3}$ and a scalar part q4∈R [25]:

$$q={\left[e^{T},q_{4}\right]}^{T}={\left[q_{1},q_{2},q_{3},q_{4}\right]}^{T}$$

and its norm equals one, that is:

∥q∥=1.

As accelerometers and gyroscopes have biases, which can be modeled as random walk processes, we have two additional vectors b and d in the state vector to represent their biases, respectively. Both variables are given within body frame B.

### 2.1. Process Model

The process model governs the dynamic relationship between the states of two successive time steps, which can be described by:

(2) $$x_{t}=f\left(x_{t-1},u_{t-1}\right)+w_{t-1}$$

where $x_{t}$ is the predicted state after time period δ based on the last known state vector $x_{t-1}$, $u_{t-1}$ is the input to the state space models and $w_{t-1}$ is the process noise. In this study, we use the following process model:

(3) $$\begin{array}{cc} x_{t} & =f\left(x_{t-1},u_{t-1}\right)+w_{t-1}\\ & =\left[\begin{array}{c} \;\;p_{t-1}+v_{t-1}\delta \;\\ \;v_{t-1}\;+\;C\;\left(q_{t-1}\right)\;\left(y_{\;a,t-1}\;-\;b_{t-1}\right)\delta \;-\;g\delta \;\;\\ \;\exp\left(\frac{1}{2}\Omega \left[y_{\omega ,t-1}-d_{t-1}\right]\delta \right)q_{t-1}\;\\ \;\left(1-1/\tau_{a}\right)b_{t-1}\\ \;\left(1-1/\tau_{\omega }\right)d_{t-1} \end{array}\right]+\;\;\left[\begin{array}{c} \;w_{p,t-1}\\ \;w_{v,t-1}\\ \;w_{q,t-1}\\ \;w_{b,t-1}\\ \;w_{d,t-1} \end{array}\right] \end{array}$$

where $u_{t-1}=\left[y_{\;a,t-1},y_{\omega ,t-1}\right]$ is the measurement vector from the accelerometer and gyroscope at time step t−1, $C\;\left(q_{t-1}\right)$ is the corresponding rotation matrix of the quaternion $q_{t-1}$ [26]:

$$C\;\left(q_{t-1}\right)=\left(q_{4,t-1}^{2}\;-\;e_{t-1}^{T}e_{t-1}\right)I_{3}+2e_{t-1}e_{t-1}^{T}-2q_{4,t-1}\;{\left[e_{t-1}\right]}_{\times }$$

representing the transformation from the body frame B to the global frame G, g is the gravitational acceleration vector and $\Omega \left[\omega \right]$ is a 4×4 skew symmetric matrix, as in:

$$\Omega \left[\omega \right]=\left[\begin{array}{cc} -{\left[\omega \right]}_{\times } & \omega \\ -\omega^{T} & 0 \end{array}\right]$$

where ${\left[\omega \right]}_{\times }$ is defined by

$${\left[\omega \right]}_{\times }=\left[\begin{array}{ccc} 0 & -\omega^{Z} & \omega^{Y}\\ \omega^{Z} & 0 & -\omega^{X}\\ -\omega^{Y} & \omega^{X} & 0 \end{array}\right]$$

where $\left[\omega {\;}^{X}\;,\omega {\;}^{Y}\;,\omega {\;}^{Z}\right]$ are the three elements of angular rates on the X-, Y- and Z-axis, respectively, and $\tau_{a}$ and $\tau_{\omega }$ are time constants. Process noise is added by the noise vector $w_{\;t-1}={\left[\begin{array}{c} w_{\;p,t-1},w_{\;v,t-1},w_{\;q,t-1},w_{\;b,t-1},w_{\;d,t-1} \end{array}\right]}^{T}$. Each noise item is modeled as a zero mean Gaussian noise with covariance matrix $Q_{p},Q_{v},Q_{q},Q_{b},Q_{d}$, respectively. The noise items are assumed to be uncorrelated with each other; thus, the process noise covariance matrix has the following expressions:

(4) $$Q=\mathrm{diag}\left[Q_{p},Q_{v},Q_{q},Q_{b},Q_{d}\right].$$

### 2.2. Measurement Model

The measurement model governs the relationship between the state vector and sensor measurements, which is:

(5) $$y_{t}=g\left(x_{t}\right)+n_{t}$$

In this paper, measurements from the GNSS receiver and encoder are introduced to bound the errors in estimates of the vehicle position/velocity and attitude. Asynchronous updates are performed within the UKF as measurements become available from the wheel encoder and GNSS receiver. For wheel encoder measurements, a filter update is calculated from the measured speed of the vehicle. For GNSS measurements, a filter update is calculated from the location of the Trimble.

#### 2.2.1. Measurement Model of GNSS

The GNSS receiver could be a differential GNSS receiver or RTK receiver, which measures data at a relatively low frequency (the measurement update rate is up to 20 Hz). When a measurement from the GNSS receiver is available, the GNSS measurement model is given by:

(6) $$y_{p}=p+n_{p}$$

where $n_{p}$ is the GNSS measurement noise modeled as a Gaussian noise, $N\;\left(0,\;\Sigma_{G\;N\;N\;S}\right)$, $y_{\;p}=C_{E}^{G}\left[p^{X}\;,p^{Y}\;,p^{Z}\right]$, and $\left[p^{X}\;,p^{Y}\;,p^{Z}\right]$ is the position on the X-, Y- and Z-axis in the ECEF frame, calculated by the following equations, respectively:

$$\begin{array}{cc} p^{X} & =\left(N+h\right)\cos\lambda \cos\phi \\ p^{Y} & =\left(N+h\right)\cos\lambda \sin\phi \\ p^{Z} & =\left[N\left(1-e^{2}\right)+h\right]\sin\lambda \end{array}$$

where $\left[\lambda ,\phi ,h\right]$ are the latitude, longitude and altitude provided by GNSS fixes. The parameters used above are defined as follows: $N=a/\sqrt{1\;-\;e^{2}\sin\lambda^{2}}$ is the length from the center of the Earth to the surface; $e=\sqrt{1\;-\;b^{2}/a^{2}}$ is the Earth eccentricity; and a= 6,378,137 (m), b= 6,356,752.3142 (m) are the Earth ellipsoid semi-major and semi-minor axes, respectively. The transition matrix from the ECEF frame to the global frame *G*, is denoted by $C_{E}^{G}$ as

$$C_{E}^{G}=\left[\;\begin{array}{ccc} -\sin\lambda \cos\phi & -\sin\lambda \sin\phi & \cos\lambda \\ -\sin\phi & -\cos\phi & 0\\ -\cos\lambda \cos\phi & -\cos\lambda \sin\phi & -\sin\lambda \end{array}\;\right].$$

The covariance matrix of the GNSS measurement noise is $R=\Sigma_{G\;N\;N\;S}$.

#### 2.2.2. Measurement Model of DMI

The measurement model for the encoder is modeled as:

(7) $$y_{v}=q^{-1}⊗v⊗q+n_{v}$$

where $y_{v}=\left[v,0,0\right]$ is the velocity of the vehicle in the body frame, *v* is the wheel speed measurement from the encoder and ⊗ represents the quaternion multiplication [25]. $n_{v}$ is the encoder measurement noise modeled as a Gaussian noise, $N\;\left(0,\;\Sigma_{Encoder}\right)$, and the covariance matrix of the encoder measurement noise is $R=\Sigma_{encoder}$.

### 2.3. Implementation of UKF

In process Model (3), due to the nonlinearity in the quaternion and velocity state functions, unscented transform-based approximation to the optimal filtering solution can be derived by executing two steps of time update and measurement update in turn. In the execution of the two steps, unscented transform is always carried out first to form the sigma points of the state vector.

#### 2.3.1. Time Update

At time step *t*, sigma points need to be calculated first and followed by performing the time update using time update equations.

- Calculate the sigma points:
  (8) $${\overset{¯}{\chi }}_{t-1}=\left[\begin{array}{cc} x_{t-1} & x_{t-1}\pm \sqrt{\left(n+\kappa \right)P_{t-1}} \end{array}\right]$$
  where ${\bar{\chi }}_{t-1}$ are the sigma points of state vector x at previous time step t−1, *n* is the dimension of the state vector x, $\kappa =\alpha^{2}\left(n+\gamma \right)-n$. α determines the spread of the sigma points and γ is a secondary scaling parameter, which is usually set to one. One should note that the initial condition $x_{0}\;∼\;N\left(x_{0},P_{0}\right)$ should be known.
- Time update process:
  (9) $${\bar{\chi }}_{t|t-1}=f\left(\chi_{t-1},u_{t-1}\right)$$
  (10) $$x_{t}^{-}=\sum_{i=0}^{2n}W_{i}^{m}{\bar{\chi }}_{i,t|t-1}$$
  (11) $$P_{t}^{-}=\sum_{i=0}^{2n}\;W_{i}^{c}\;\left({\bar{\chi }}_{i,t|t-1}\;-\;x_{t}^{-}\;\right){\left({\bar{\chi }}_{i,t|t-1}\;-\;x_{t}^{-}\;\right)}^{T}+\;Q$$
  where $x_{t}^{-}$ and $P_{t}^{-}$ are the predicted mean and covariance, respectively, and $W_{i}^{m}$ and $W_{i}^{c}$ are the weights of mean and covariance, which are associated with the *i*-th point, given by [27]:
  $$\begin{array}{cc} W_{0}^{m} & =\frac{\kappa }{n+\kappa }\\ W_{0}^{c} & =\frac{\kappa }{n+\kappa }+\left(1-\alpha^{2}+\beta \right)\\ W_{i}^{m} & =W_{i}^{c}=\frac{1}{2(n+\kappa )},i=1,2,...,2n \end{array}$$
  where β is a parameter used to incorporate any prior knowledge about the distribution of state x (for Gaussian distributions, β=2 is optimal).

#### 2.3.2. Measurement Update of GNSS

When fix measurement $y=y_{\;p}$ is available, we can update the nearest state prediction $x_{t}^{-}$ and covariance matrix $P_{t}^{-}$ using the following equations. At first, sigma points need to be calculated and then, measurement update is performed using measurement update equations.

- Calculate the sigma points:
  (12) $${\bar{\chi }}_{t}=\left[\begin{array}{cc} x_{t}^{-} & x_{t}^{-}\pm \sqrt{\left(n+\kappa \right)P_{t}^{-}} \end{array}\right]$$
  where $x_{t}^{-}$ and $P_{t}^{-}$ are the predicted mean and covariance from time update at time *t*, respectively.
- Perform measurement update:
  (13) $${\bar{Y}}_{t}=g\left({\bar{\chi }}_{t}\right)$$
  (14) $${\bar{y}}_{t}=\sum_{i=0}^{2n}W_{i}^{m}{\bar{Y}}_{i,t}$$
  (15) $$P_{y_{t}}=\sum_{i=0}^{2n}W_{i}^{c}\left({\bar{Y}}_{i,t}-{\bar{y}}_{t}\right){\left({\bar{Y}}_{i,t}-{\bar{y}}_{t}\right)}^{T}+R$$
  (16) $$P_{x_{t}y_{t}}=\sum_{i=0}^{2n}W_{i}^{c}\left({\bar{\chi }}_{i,t}-x_{t}^{-}\right){\left({\bar{Y}}_{i,t}-{\bar{y}}_{t}\right)}^{T}$$
  (17) $$K_{t}=P_{x_{t}y_{t}}{\left(P_{y_{t}}\right)}^{-1}$$
  (18) $$\nu_{t}=y_{p,t}-{\bar{y}}_{t}$$
  (19) $$x_{t}=x_{t}^{-}+K_{t}\nu_{t}$$
  (20) $$P_{t}=P_{t}^{-}-K_{t}P_{y_{t}}K_{t}^{T}$$
  where ${\bar{Y}}_{t}$ are the projected sigma points through the measurement function *h*, ${\bar{y}}_{t}$ is the predicted measurement produced by the weighted sigma points, $P_{\;\;y_{t}}$ and $P_{\;\;x_{t}y_{t}}$ are the predicted measurement covariance and the state-measurement cross-covariance matrix, respectively, $K_{t}$ is the Kalman gain, $\nu_{t}$ is the innovation and $x_{t}$ and $P_{t}$ are the updated state and covariance at time *t*, respectively.

#### 2.3.3. Measurement Update of DMI

When wheel speed measurement $y=y_{\;v}$ is available, we can update the nearest state prediction $x_{t}^{-}$ and covariance matrix $P_{t}^{-}$ using the similar equations with fix measurement update. Similarly, sigma points need to be calculated, and then, measurement update is performed using measurement update equations.

### 2.4. Automatic Detection the Degradation of GNSS Performance

GNSS suffers multi-path errors if the satellite signals are reflected off one or more surfaces before reaching the receiver antenna. A different set of satellites is utilized for fix determination, which can also alter the GNSS fix. Big gaps occur when GNSS signals become available again after short-term GNSS dropouts due to the presence of trees and buildings. Different checks are adopted to make our pose estimator robust to the aforementioned jumps, which are explained as follows:

Zero-Velocity update: As GNSS receivers due to pseudo-random error cannot output fixed position information when the vehicle stays in a stationary position, if the vehicle is not in motion, which can be well detected from the encoder readings, measurements from the receiver are not used for updates. This will restrict the vehicle to a fixed location.

Number of satellites: If the number of satellites visible to the receiver is four or more, the measurements from the receiver pass the check.

Dilution Of Precision (DOP): If Horizontal DOP (HDOP) or Vertical DOP (VDOP) is larger than a threshold, the measurements will be discarded.

Statistical test: During the GNSS measurements update, if an abrupt jump in the GNSS fix occurs, the correction made by the GNSS measurement update will cause the IMU solutions to incorrectly follow these jumps. The chi-squared test is applied once the innovation ν and the innovation covariance matrix $\Sigma_{\nu }$ are obtained in the measurement update [28], as in:

(21) $$\nu^{T}\Sigma_{\nu }^{-1}\nu \leq \varsigma$$

where the value ς is usually set to reject the innovations exceeding the 95% threshold. During the GNSS measurement update stage, if (21) holds, then the GNSS fix is accepted, and the measurement update proceeds.

Assessing the new horizontal position reading and subtracting it from the current estimate of position: If the difference is much higher than what it should be when compared to the vehicle speed obtained from the encoder, which is assumed to be an accurate quantity, then the measurements are discarded.

Check the altitude component: If the measurements are much higher than the innovation, the updates will be aborted.

Validity of position change: After the GNSS measurement update, the change in position Δp can be calculated by:

(22) $$\Delta p=p_{t}-p_{t-1}$$

The change in position is invalid if it satisfies:

(23) $$\left|\Delta p\right|\;>\;v\left(1+\eta \right)\;\Delta t+\epsilon \vee \left(\left|\Delta p\right|\;>\;\epsilon \wedge \frac{\Delta p}{\left|\Delta p\right|}\;\cdot \;\left[\begin{array}{c} \;\cos\psi \\ \;\sin\psi \end{array}\right]\;>\;\tau \;\right)$$

where ψ represents the travel heading, calculated from quaternion q [25]. η, ϵ and τ are three constants representing the anticipated percentage velocity error, allowed position jitter and allowable travel direction error, respectively. When Δp is rejected, a predicted position is calculated based on heading and wheel speed.

The accuracy of the estimated location of the vehicle is about 1–3 cm or 1 m depending on RTK or if the differential mode is operating.The resulting lateral offset will not guarantee safe driving for autonomous vehicles. In the following section, we are going to introduce the lateral localization based on LiDAR measurements to improve the accuracy of the localization results.

## 3. Correction of Lateral Localization Errors

After we get the localization result from the fusion of GNSS/IMU/encoder, the lateral localization error can be calculated as follows. The detection of curbs using LiDAR (Light Detection And Ranging) provides an accurate lateral distance between the vehicle and curb (denote it by $d_{1}$); at the same time, the same distance can also be calculated using the localization result from GNSS/encoder and RNDF (Route Network Definition File) information (denote it by $d_{2}$). The difference of $d_{1}$ and $d_{2}$, i.e., $d_{1}-d_{2}$, is the lateral localization error, which can readily be corrected.

**Figure 2:**
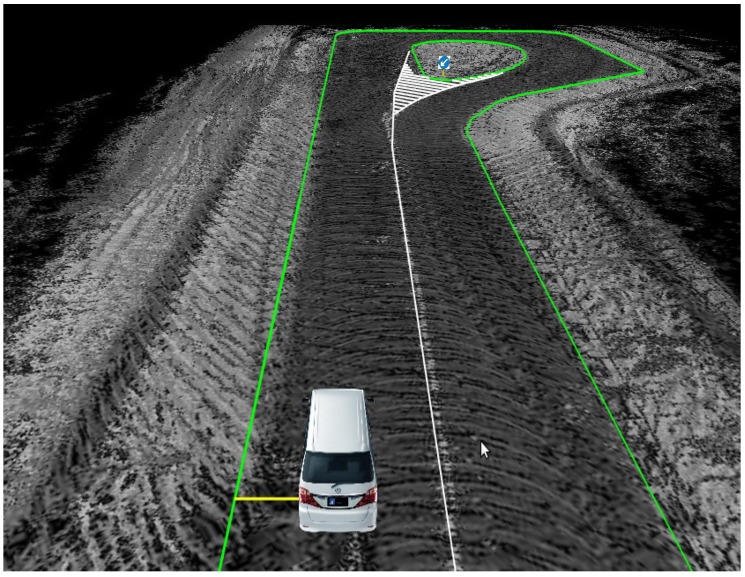
Lateral distance from the vehicle to the curb.

Assume that the RNDF (Route Network Definition File) information is accurate enough: given the vehicle position provided by the GNSS/IMU/DMI fusion system, a lateral distance from the vehicle to the curb can always be obtained through a simple geometry calculation; see the yellow line segment in Figure 2.

**Figure 3:**
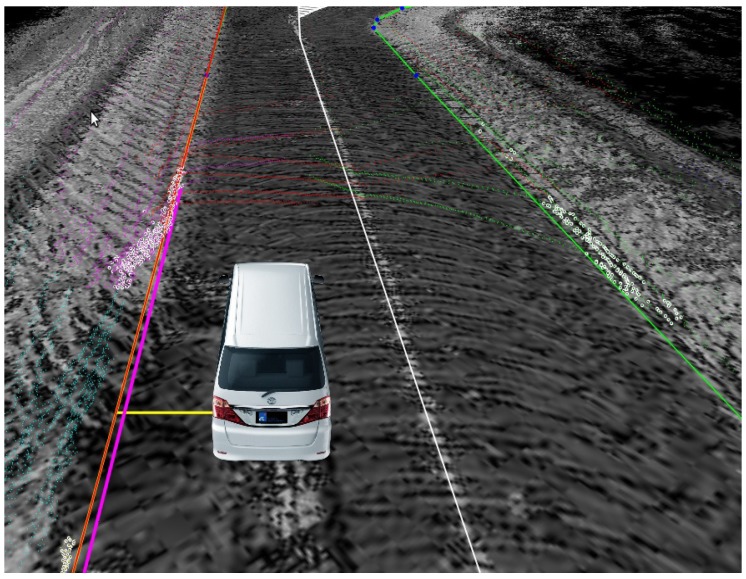
Curb estimated by fitting.

However, as known to all, the result of the GNSS/IMU/DMI fusion system is always affected by drifts due to the signal failure of GNSS caused by the complexity of the urban environment. This drift may cause the vehicle to hit the curb or rush to the next lane, which may cause a serious accident. In Figure 3, the pink line is the curb line that is fitted by the point cloud collected by a 3D LiDAR, whose accuracy is less than 10 cm, and the orange line is the curb line of the RNDF; obviously, there is a large difference between the vehicle to these two lines, which is caused by the drift of the localization result. Thus, a lateral correction is very necessary for the localization of the autonomous vehicle. To accomplish the goal of LiDAR-based lateral error correction, we first detect longitudinal curbs and use them as measurements to estimate the autonomous vehicle’s lateral distance, then compare it with the distance calculated from RNDF, e.g., the yellow line as shown in Figure 2. The difference of these two distances is then used to correct the lateral error of the autonomous vehicle.

### 3.1. Curb Detection

#### 3.1.1. Curb Detection Principle

**Figure 4:**
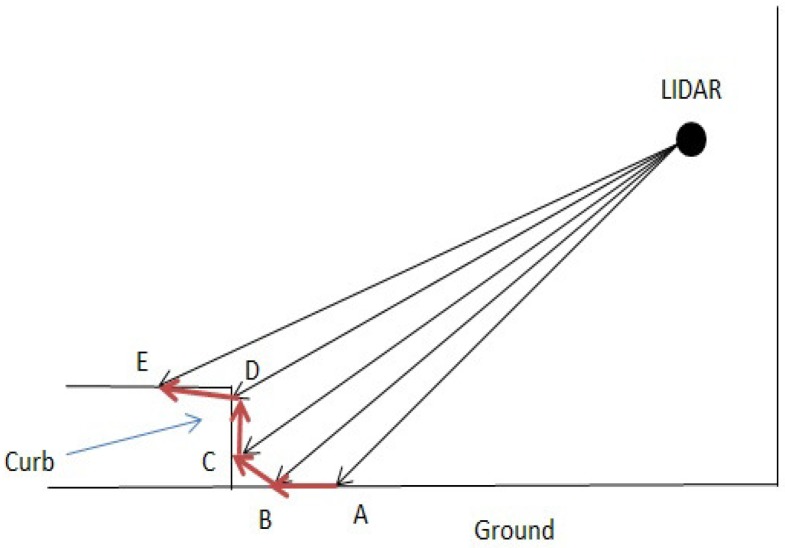
Points selected on the curb. A,B,C,D are 3D points obtained by LiDAR. *B* and *C* are selected as points on the curb since the vectors $\vec{BC}$ and $\vec{CD}$ are more “upcast”.

The first step for lateral correction is the curb detection. Figure 4 shows the curb detection principle. Assume that A,B,C,D,E are part of the adjacent points collected by one beam of a 3D LiDAR; the vectors $\vec{AB}$, $\vec{BC}$, $\vec{CD}$, $\vec{DE}$ can be calculated, and B,C can be selected as the curb point since the angle between $\vec{BC}$ and the ground is very large, the same for vector $\vec{CD}$.

#### 3.1.2. Algorithm for Curb Detection

This subsection provides Algorithm 1 which is used for curb detection.

**Figure 5:**
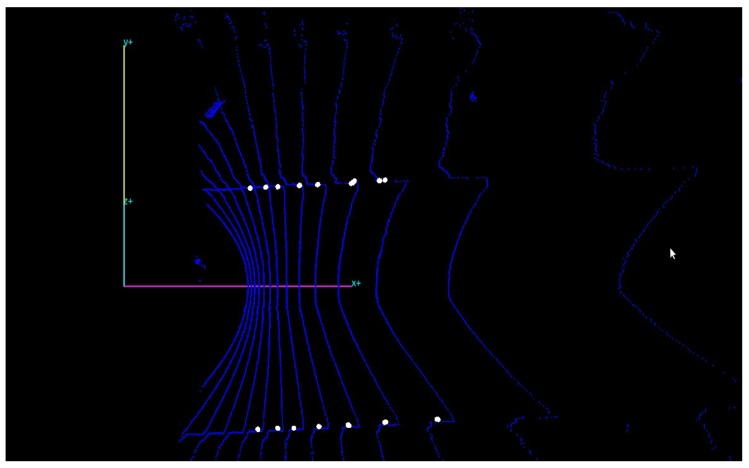
Output to the curb detection module.

The following Figure 5 presents an example of the curb detection result where curb points are marked as white points.
**Algorithm 1** Framework of curb detection.**Require:**
 Point clouds collected by a 3D LiDAR;
**Ensure:**
 Step 1: Given input point cloud, select the area of interest;
 Step 2: Calculate the vector difference of adjacent points in each beam;
 Step 3: Select curb-like points and filter out noises;
 Step 4: Separate higher obstacles by comparing the height with a threshold;
 **return** Curb points.

### 3.2. Curb Line Fitting Using RANSAC

After detecting the curb, the next step is to fit the curb line using the RANSAC algorithm, which is an iterative method to estimate the parameters of a curve from detected curb points. The details are presented in the following Algorithm 2.

Denote *S* as a set of curb points that are separated from higher obstacles, then we have

**Algorithm 2** Framework of curb fitting.**Require:**
 Detected curb points;
**Ensure:**
 Step 1: Randomly select a sample of *s* curb points from *S*, and instantiate the model from this subset;   Step 2: Determine the set of curb points $S_{i}$ that are within a distance threshold *t* of the model. The set $S_{i}$ is the consensus set of samples and defines the inliers of *S*;  Step 3: If the subset of $S_{i}$ is greater than some threshold *T*, re-estimate the model using all of the points in $S_{i}$ and terminate;   Step 4: If the size of $S_{i}$ is less than *T*, select a new subset and repeat the above;   Step 5: After *N* trials, the largest consensus set $S_{i}$ is selected, and the model is re-estimated using all of the points in the subset $S_{i}$.
 **return** Curb model.

In Algorithm 2, the calculation of the distance is a key point, since there are two curb cases: straight line and curve; the distance calculation is different for these two cases. Fortunately, given the curb map obtained offline and the vehicle position, the shape of the curb can be known beforehand since the curb shape does not change much within a certain area. The following are the individual methods to calculate the distance.

**Figure 6:**
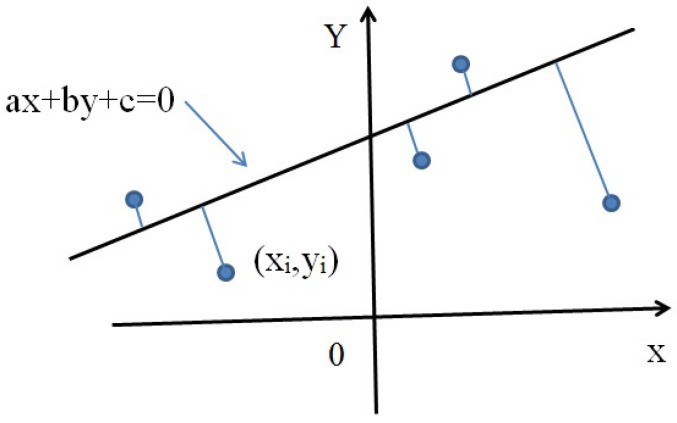
Straight line curb.

**A. Straight line curb case:** In Figure 6, the positive direction of the X coordinate denotes the heading direction of the vehicle; the positive direction of the Y coordinate denotes the left side of the vehicle; the straight line denotes the curve, and its algebraic expression is:

(24) $$\begin{array}{c} ax+by+c=0 \end{array}$$

assume the coordinate of one curb point is $\left(x_{i},y_{i}\right)$ and the distance $d_{i}$ is defined as the minimum distance from $\left(x_{i},y_{i}\right)$ to the straight line, which can be calculated as:

(25) $$\begin{array}{c} d_{i}=\frac{|ax_{i}+by_{i}+c|}{\sqrt{a^{2}+b^{2}}} \end{array}$$

**Figure 7:**
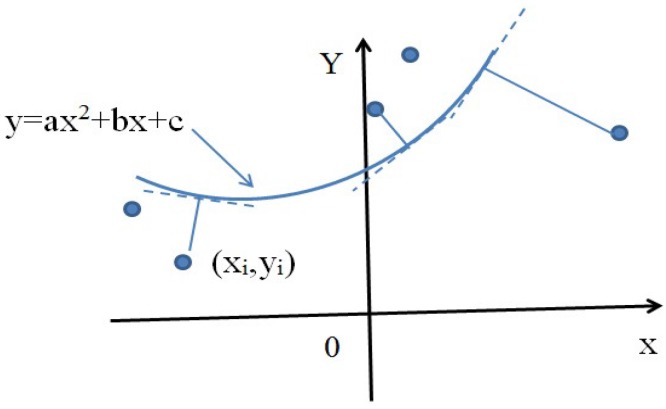
Curve line curb.

**B. Curve line curb case:** Similar to Figure 6, in Figure 7, the positive direction of the X coordinate denotes the heading direction of the vehicle; the positive direction of the Y coordinate denotes the left side of the vehicle. The curve line denotes the curb, and its algebraic expression is:

(26) $$\begin{array}{c} y=ax^{2}+bx+c \end{array}$$

In addition, each dashed line denotes the tangent line of the curve, which is perpendicular to the line joining the point of tangency and the curb point $\left(x_{i},y_{i}\right)$. Assume that the coordinate of one curb point is $\left(x_{i},y_{i}\right)$; the distance $d_{i}$ is also defined as the minimum distance from $\left(x_{i},y_{i}\right)$ to the curve, which can be calculated as follows:

Firstly, we need to find one point on the curve that is perpendicular to the line joining the point of tangency and the curb point $\left(x_{i},y_{i}\right)$. Denote this point as $\left(x,ax^{2}+bx+c\right)$; the slope of the tangent line across this point can be calculated as:

$$\begin{array}{c} k_{1}=2a+b \end{array}$$

and the slope joining this point and the curb point $\left(x_{i},y_{i}\right)$ is:

$$\begin{array}{c} k_{2}=\frac{y_{i}-\left(ax^{2}+bx+c\right)}{x_{i}-x} \end{array}$$

From $k_{1}k_{2}=-1$, we have:

(27) $$\begin{array}{c} A_{i}x^{3}+B_{i}x^{2}+C_{i}x+D_{i}=0 \end{array}$$

where:

$$\begin{array}{c} A_{i}=2a^{2},\\ B_{i}=3ab\\ C_{i}=2ac+b^{2}+1-2ay_{i},\\ D_{i}=bc-by_{i}-x_{i} \end{array}$$

The real root of (27) is:

(28) $$\begin{array}{c} x^{*}=-\frac{1}{3A_{i}}\left(B_{i}+C_{i}+\frac{\delta_{0}}{\delta_{2}}\right) \end{array}$$

where:

$$\begin{array}{c} \delta_{0}=B_{i}^{2}-3A_{i}C_{i}\\ \delta_{1}=2B_{i}^{3}-9A_{i}B_{i}C_{i}+27A_{i}^{2}D_{i}\\ \delta_{2}={\left(0.5\left(\delta_{1}+\sqrt{\delta_{1}^{2}-4\delta_{0}^{3}}\right)\right)}^{\frac{1}{3}} \end{array}$$

Then, we have:

(29) $$\begin{array}{c} y^{*}=a{x^{*}}^{2}+bx^{*}+c \end{array}$$

The minimum distance from curb point $\left(x_{i},y_{i}\right)$ to the curve is:

(30) $$\begin{array}{c} d_{i}=\sqrt{{\left(x_{i}-x^{*}\right)}^{2}+{\left(y_{i}-y^{*}\right)}^{2}} \end{array}$$

The following Algorithm 3 presents the pseudo-code of curb fitting.

**Algorithm 3** Pseudo-code of curb fitting.**Require:**
 *M* 3D points (only the X and Y coordinates are used);
**Ensure:**
 Step 1: Initialize parameters of the algorithm. Let N=1000; T=0.6×M;  Step 2: Repeat for *N* iterations:   a. Select two points randomly from the *M* points;   b. Compute the parameters (a,b,c) that define the line passing through those two points;   c. Count the number of inliers for the current line;  d. If the number of inliers is greater than or equal to *T*, terminate;  e. Keep the line having a maximum number of inliers;  Step 3: Draw the line having the maximum number of inliers.  **return** Line/curve parameters of curbs.

### 3.3. Lateral Correction Based on the Kalman Filter

#### 3.3.1. Lateral Error Estimation

**Figure 8:**
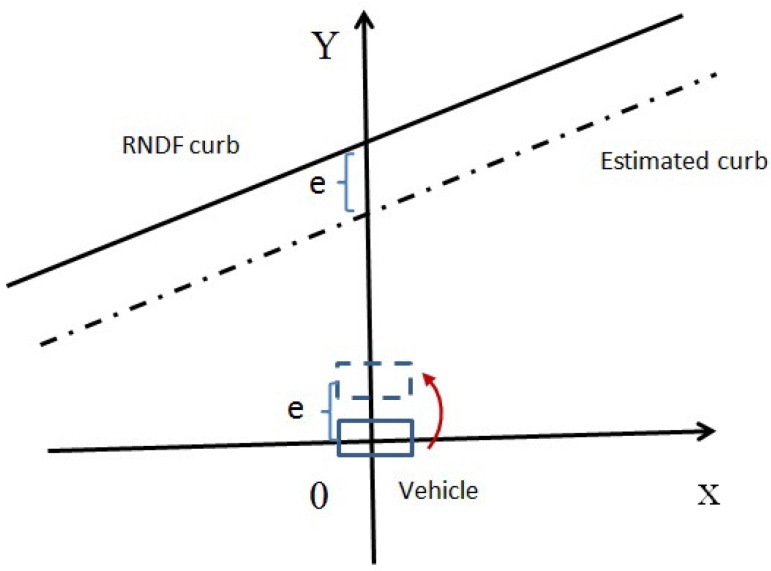
Lateral distances.

After fitting the curb into straight lines or curves (roundabout area) by using the candidate points selected from the point cloud of a 3D LiDAR, the next step is to calculate the lateral distance from the vehicle to the curb. Assume the curb is straight. The curve case is similar, as shown in Figure 8. The solid line denotes the RNDF curb. The dash-dotted line denotes the curb estimated. The origin denotes the position of the vehicle. In the lateral direction, i.e., the positive direction of the Y coordinate, there is a gap e, which is the lateral error caused by the localization error. Obviously, in order to correct this error, we need to adjust the vehicle position from the origin to the position of the rectangle on the dashed line, which means move the vehicle a distance e along the positive direction of the Y coordinate.

In order to filter the noise caused by sensor and measurement noise, etc., and smooth the lateral adjustment, a one-dimensional Kalman filter [29] is used to estimate the difference of lateral distances from the vehicle to the estimated curb and RNDF curb. Define the measurement vector $y_{t}$ as the difference e and the state vector $x_{t}$ as the systematic part of this difference. Assume the change of *x* in time to be random. Set the system transition matrix as the identity matrix. We have that the system of equation takes the form:

(31) $$\begin{array}{c} x_{t}=x_{t-1}+w_{t} \end{array}$$

the observation equation is given by:

(32) $$\begin{array}{c} y_{t}=x_{t}+v_{t} \end{array}$$

where $w_{t},v_{t}$ are scalar variables of zero mean. The initial value $x_{0}$ is assumed to be zero. Then, our Kalman filter algorithm has the following form:

- State space: The space of real number R
- State Vector: $x_{t}$
- System equation: $x_{t}=x_{t-1}+w_{t}$
- Observation: $y_{t}=x_{t}+v_{t}$
- Prediction equation: $x_{t/t-1}=x_{t},P_{t/t-1}=P_{t-1}+W_{t}$
- Updating equations: $x_{t}=x_{t/t-1}+K_{t}\left(y_{t}-x_{t/t-1}\right)$,
  $$K_{t}=\frac{P_{t/t-1}}{P_{t/t-1}+V_{t}}$$
  $$P_{t}=\left(1-K_{t}\right)P_{t/t-1}$$

#### 3.3.2. Lateral Adjustment

After we obtain the lateral difference e, which is the output of the one-dimensional Kalman filter as stated in the last subsection, next, we need to calculate the adjustment of the vehicle position in the global frame. Here we denote the adjustment as (Δx,Δy).

Assume the global pose of the vehicle is $\left(x_{v},y_{v},\phi_{v}\right)$, and the intersection point coordinate of the estimated curb line and Y coordinate in the local coordinate frame of the vehicle is $\left(x_{l},y_{l}\right)$, while the global coordinate of this intersection point is denoted as $\left(x_{g},y_{g}\right)$, then we have:

$$\begin{array}{c} \Delta x=\frac{e}{d_{RNDF}}\left(x_{g}-x_{v}\right)\\ \Delta y=\frac{e}{d_{RNDF}}\left(y_{g}-y_{v}\right) \end{array}$$

where *e* is the lateral distance error, $d_{RNDF}$ is the lateral distance from vehicle to the RNDF curb line and:

$$\begin{array}{c} x_{g}=x_{v}+x_{l}\cos\left(\phi_{v}\right)-y_{l}\sin\left(\phi_{v}\right)\\ y_{g}=y_{v}+x_{l}\sin\left(\phi_{v}\right)+y_{l}\cos\left(\phi_{v}\right) \end{array}$$

Then, (Δx,Δy) are added to the vehicle global position to correct the lateral error, i.e.,

$$\begin{array}{c} x_{v}^{\prime }=x_{v}+\Delta x\\ y_{v}^{\prime }=y_{v}+\Delta y \end{array}$$

where $\left(x_{v}^{\prime },y_{v}^{\prime }\right)$ is the vehicle coordinate after adjustment, which corresponds to the $\left[p^{X}\;,p^{Y}\;\right]$ of the vehicle position p in (1).

## 4. Experimental Results

### Result of GNSS/INS/DMI-Based Localization

In this subsection, we evaluate the performance of GNSS/IMU/DMI-based localization quantitatively using the publicly available dataset proposed in [30], where the ground truths of poses are provided to be 1.0 cm and 0.5${}^{\circ }$. Since the dataset does not include encoder data, artificial encoder data are generated from the RTK GNSS speed measurements, which are used as the ground truth. They are upsampled to get the output rate of 100 Hz. The proposed approach is tested on the experimental Campus-0L (5.563 min) in which the trajectory totally covers about 1143 m in distance with two sharp turns. In this paper, we compare the pose estimates from two UKF-based methods with the reference: one is the UKF method with the jump detection strategies, represented by ‘UKF’; the other one is the UKF method without jump detection involved, represented by ‘UKF w/o’. In what follows, we use ‘REF’ to represent the reference trajectory.

**Figure 9:**
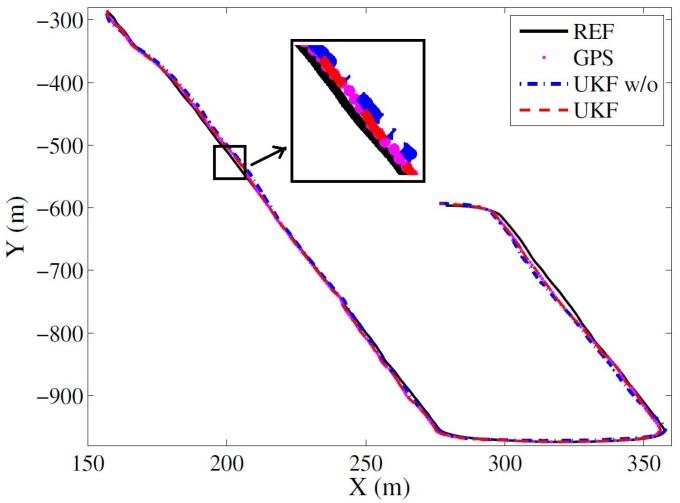
The estimated trajectories by the UKF-based methods with or without (w/o) the jump detection strategies against the reference.

Figure 9 illustrates the estimated vehicle trajectories in the horizontal plane provided by the two methods against the reference. In the plot, the solid black line represents the reference trajectory; the dots indicate the GNSS measurements; the red dashed line shows the estimated trajectory by ‘UKF’; and the blue dash-dotted line demonstrates the trajectories estimated by ‘UKF w/o’. As we can see from the figure, the estimated trajectories from both methods follow the reference well, as there is no obvious jumps in this dataset.

**Figure 10:**
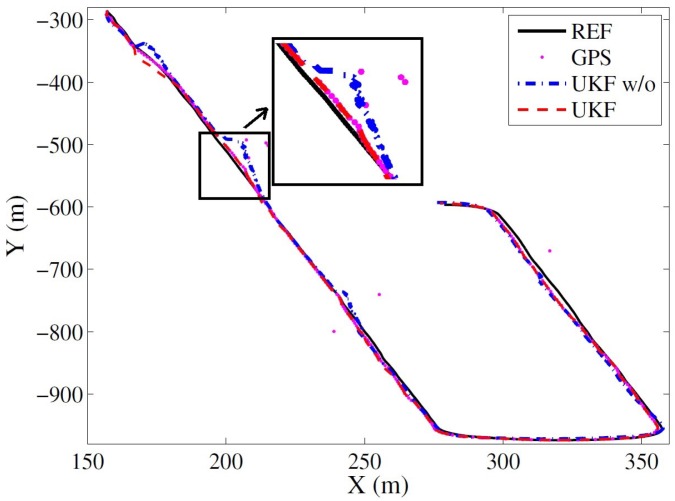
The estimated trajectories by the UKF-based methods with or without the jump detection strategies with random noises in the GNSS measurements against the reference.

During this experiment, no jump exists in the DGNSSmeasurements. To verify the efficiency of jump detection, some random noises were intentionally induced, which can be seen from the GNSS measurements represented by dots in Figure 10. Figure 10 illustrates the estimated trajectories in the horizontal plane against the reference with simulated GNSS jumps. The results are provided by the ‘UKF w/o’ and ‘UKF’ methods. As we can see from the figure, even with the GNSS jumps, the trajectory estimated by the ‘UKF’ method still follows the reference very well; however, the trajectory from the ‘UKF w/o’ method deviates from the reference when GNSS jumps are introduced.

**Figure 11:**
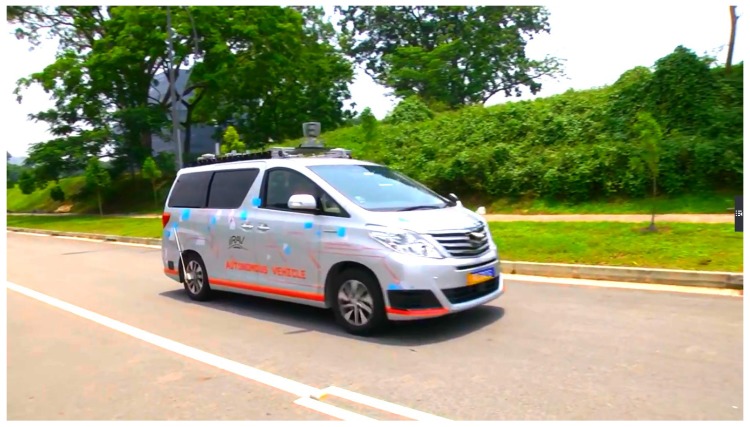
Autonomous vehicle equipped with GPS/IMU/DMI/LiDAR.

From Figure 9 and Figure 10, it can be seen that though the accuracy of position using UKF has been improved, lateral localization errors still exist, which may need to be corrected. To verify the lateral error correction method proposed in this paper, we apply it to the autonomous vehicle of the Institute for Infocomm Research, Agency for Science, Technology and Research (A*STAR), Singapore, which is equipped with GPS/IMU/DMI/LiDAR [31]; see Figure 11.

**Figure 12:**
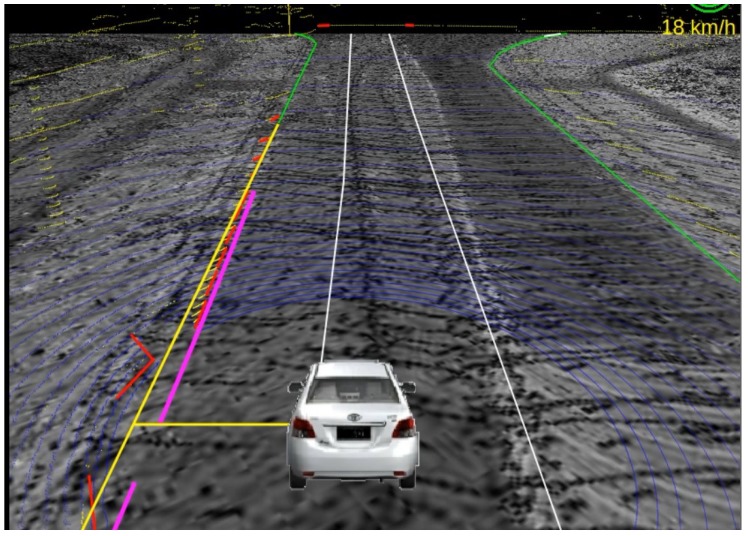
Localization result without lateral correction.

**Figure 13:**
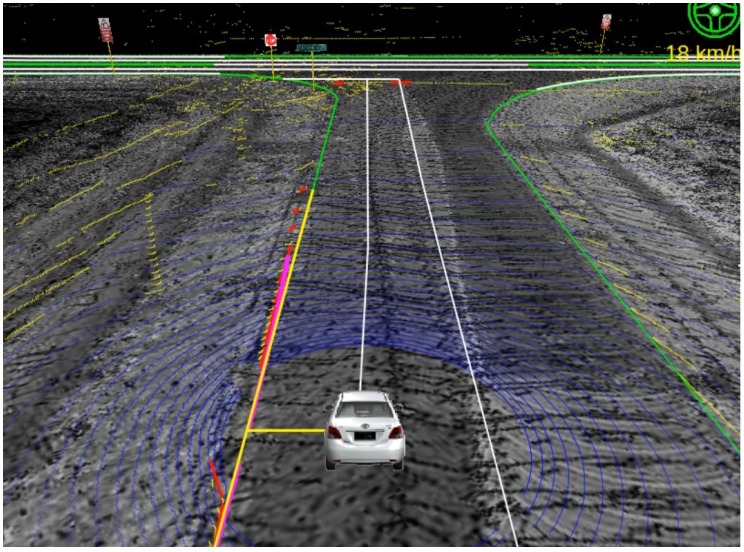
Localization result with lateral correction.

**Figure 14:**
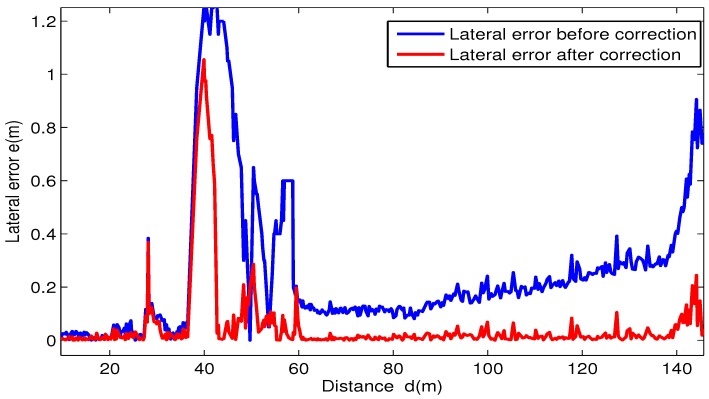
Lateral errors before and after lateral correction.

Point clouds of curb-like obstacles around the vehicle are detected by the 3D LiDAR mounted on the vehicle. After separating curbs from other obstacles and fitting them into straight lines and curve lines, lateral distances from vehicle to the curbs are calculated, then we apply the lateral correction method proposed in Section 3 to correct the lateral error of the GPS/IMU/DMI localization results. Figure 12 and Figure 13 show the localization results of the autonomous vehicle without and with lateral error correction, respectively, where pink lines denote the curb detected using the method proposed in this paper, and the long yellow line segment in each figure is the curb line obtained from RNDF, while the short yellow line segment denotes the lateral distance between the vehicle to RNDF curbs. Figure 14 shows the lateral errors before and after lateral correction during a period of time. From Figure 12, Figure 13 and Figure 14, it can seen that lateral localization errors have been improved greatly.

## 5. Conclusions

In this paper, to further improve the localization accuracy of an autonomous vehicle using the UKF-based GNSS/IMU/DMI fusion method, a multi-constraint fault-detection approach is proposed to handle the GNSS jumps. In addition, point cloud-based lateral correction is also proposed, where curbs are estimated in real time using a 3D LiDAR. A one-dimensional Kalman filter is adopted to estimate the lateral errors, which are used to improve lateral localization. Our method suppresses falsely-detected curb points by using the RANSAC algorithm. In future work, after the selection of point clouds for curbs, machine learning and deep learning algorithms may be used to further process the point clouds, such that curbs can be separated from other obstacles, such as vehicles and pedestrians.
